# Retrieval of lost patients in the system for hepatitis C microelimination: a single-center retrospective study

**DOI:** 10.1186/s12876-021-01792-8

**Published:** 2021-05-08

**Authors:** Hsu-Heng Yen, Pei-Yuan Su, I.-L.ing Liu, Ya-Huei Zeng, Siou-Ping Huang, Yu-Chun Hsu, Po-Ke Hsu, Yang-Yuan Chen

**Affiliations:** 1 Division of Gastroenterology, Department of Internal Medicine, Changhua Christian Hospital, No. 135 Nanhsiao Street, Changhua, Taiwan; 2 General Education Center, Chienkuo Technology University, Changhua, Taiwan; 3 Department of Electrical Engineering, Chung Yuan Christian University, Taoyuan, Taiwan; 4 Department of Hospitality Management, MingDao University, Changhua, Taiwan

## Abstract

**Background:**

Hepatitis C virus (HCV) is one of the major causes of chronic liver disease, cirrhosis, and liver cancer. Most of the infected people have no clinical symptoms. The current strategy for HCV elimination includes test and treatment. In this study, we aimed to evaluate the campaign for retrieving patients who were lost to follow-up, for subsequent re-evaluation.

**Methods:**

From January 2020 to October 2020, patients who had prior tests for positive anti-HCV antibody in 2010–2018 in our hospital were enrolled for our patient callback campaign. Patients who had unknown HCV RNA status or no documented successful antiviral therapy history were selected for anti-HCV therapy re-evaluation. To facilitate patient referral in the hospital, we developed an electronic reminding system and called the candidate patients via telephone during the study period.

**Results:**

Through the hospital electronic system, 3783 patients with positive anti-HCV antibody documentation were identified. Among them, 1446 (38.22%) had tested negative for HCV RNA or had anti-HCV therapy, thereby excluded. Of the 2337 eligible patients, 1472 (62.99%) were successfully contacted and called back during the study period for subsequent HCV RNA testing and therapy. We found that 42.19% of the patients had positive HCV RNA and 88% received subsequent anti-HCV therapy.

**Conclusions:**

A significant number of patients with positive HCV serology were lost for HCV confirmatory test or therapy in the hospital. Therefore, this targeted HCV callback approach in the hospital is feasible and effective in achieving microelimination.

## Introduction

Chronic hepatitis C (CHC) is a major cause of chronic liver disease and liver cancer worldwide. Currently, with the newly developed direct-acting antiviral agents (DAAs), treatment of hepatitis C virus (HCV) infection has revolutionized compared with that during the interferon-based era, with significantly high rates of sustained virologic response (SVR) (> 90%) and good treatment tolerance even in those groups that are difficult to treat [1–4]. Through screening for early diagnosis and reducing the barriers for HCV treatment, the prevalence of HCV-induced chronic liver diseases and HCV-related mortality will be mitigated. The screening and diagnosis of HCV infection and patients’ adherence to treatment involve several barriers, including cost, patient awareness, and in adequate manpower. The microelimination approach [5, 6] in specific populations is less complex and less costly than the nationwide elimination approach. Specific high-risk populations such as injection drug users [7], prisoners [8], HIV-infected patients [2, 9], and dialysis patients [3] have been screened as the first step toward HCV elimination.

One target population for HCV elimination is those patients with records of hepatitis C antibody testing in the hospital. Testing for hepatitis C can be performed for several reasons, such as survey for abnormal liver function, preoperative checkup, blood donation, before chemotherapy, or routine physical checkup.

Despite that HCV antibody–positive patients clearly need HCV testing or treatment, Fujii [10] reported the lack of intrahospital collaboration even in medical institutions with hepatologists. Only 12% of probable HCV-positive patients were referred to specialists [11], and the majority were lost to follow-up; thus, they did not receive the subsequent therapy. Given that these new DAAs have been reimbursed by Taiwan health system since 2017 and the government set a goal of 80% treatment coverage rate with DAAs by 2025 in Taiwan [12], our hospital organized the “Retrieval of Lost Patients of the Hospital” strategy in 2020, aimed to eliminate HCV infection in the hospital by searching first for patients with a history of HCV infection who were lost to follow-up. Thus, the current study aimed to evaluate the outcome of this hospital-based HCV elimination approach for patients who were lost to follow-up.

## Materials and methods

In 2020, the Health Promotion Administration of Ministry of Health and Welfare in Taiwan initiated a hepatitis C testing and treatment program that aims to eliminate HCV infection nationwide. As the only medical center in Changhua County, with an area of 1074 km^2^ and a population of 1.2 million located in central Taiwan, our hospital organized a hospital-based project called “Retrieval of Lost Patients of the Hospital” in response to the hepatitis C elimination campaign established by the Taiwan government in 2020.

Two dedicated nurses (Liu IL and Zang YH) recontacted patients who were eligible for re-evaluation and one senior hepatologist (Su PY) reviewed the patients’ records to evaluate the recent status of patients’ survival and comorbidity status. Furthermore, using PharmaCloud in Taiwan [13], we could assess the pharmacy record of patients in the last 3 months to determine if they had been treated in other hospitals. Subsequent strategies included posting reminding sticks or electronic messages during clinic visits to facilitate referring patients to our hepatologist for subsequent evaluation and contacting the patients via telephone, SMS, or mails as possible. For HCV RNA testing and patient referral, this callback campaign adopted an HCV reflex test [14] approach with simultaneous HCV RNA and genotype checkup.

Patients who tested positive for hepatitis C antibody in the hospital between 2010 and 2018 were included for the analysis. Under the approval by Changhua Christian Hospital Institutional Review Board (CCH IRB No. 200403) with the consideration of the retrospective design of the study, informed consent was waived. From the electronic medical records, medical information regarding patients’ demographics, comorbid conditions, anti-HCV treatment history, and laboratory values for HCV RNA status was extracted. All procedures conformed to the guidelines and regulations of Changhua Christian Hospital.

### Statistical analysis

Data are expressed as n/N (%), median (interquartile range), or means ± standard deviation. The distribution of continuous variables was examined by one-sample Kolmogorov–Smirnov test. We used the chi-square test or the Fisher’s exact test for comparing categorical variables and the Mann–Whitney *U* test or Student’s *t*-test for comparing continuous variables. All statistical data were analyzed using the SPSS software version 18.0 (SPSS Inc., Chicago, IL, USA), with p < 0.05 indicating statistical significance.

## Results

### Demographic characteristics of the study population

**Fig. 1:**
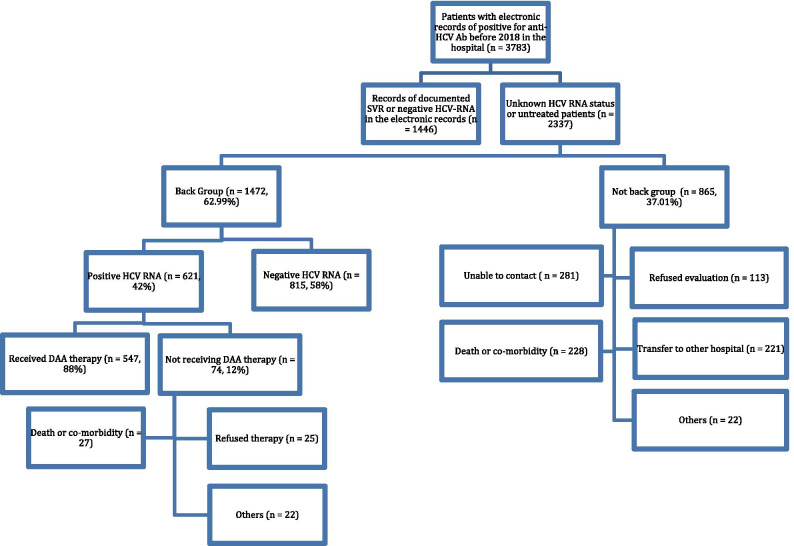
Patient flow chart

**Fig. 2:**
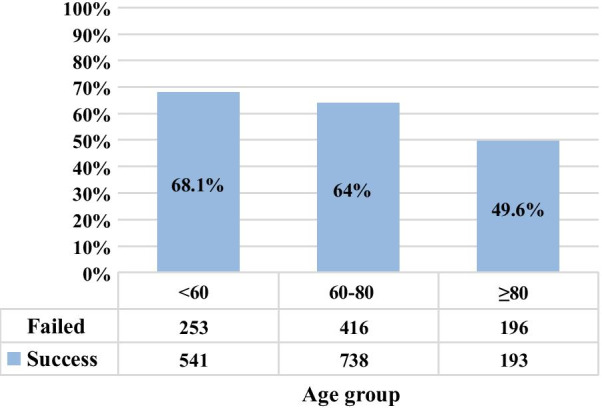
Patient retrieval rate according to age group

Figure 1 illustrates the study’s patient enrollment flow. We identified 3783 patients with a record of positive anti-HCV antibody test from the hospital laboratory system. However, 1446 of them had a documentation of negative HCV RNA or SVR from anti-HCV therapy, thereby excluded from the analysis. Ultimately, we analyzed the remaining 2337 patients; their demographic characteristics are summarized in Table 1. Most of the patients aged 60–80 years (Fig. 2). Patients who were younger (< 60), were male, and recently visited the gastroenterology division or internal medicine department as an outpatient had a higher success rate of callback. Conversely, patients who underwent clinic visits in departments other than the surgery or internal medicine department or aged > 80 years had a lower callback success rate. Of the 1472 (42.2%) patients tested for HCV RNA, 621 were positive.

**Table 1:** Comparison of characteristics of patients with successful or failed callback for HCV testing

	ALL patients (n = 2337)	Back (n = 1472)	Not Back (n = 865)	p value
*Age, year, median (IQR)*	65 (55–75)	64 (54–73)	67 (57–78)	< 0.001
*Age level, n/N (%)*				< 0.001
< 60	794/2337 (34.0)	541/1472 (36.8)	253/865 (29.2)	< 0.001
60–80	1154/2337 (49.4)	738/1472 (50.1)	416/865 (48.1)	0.362
≥ 80	389/2337 (16.6)	193/1472 (13.1)	196/865 (22.7)	< 0.001
*Gender (M), n/N (%)*	1151/2337 (49.3)	762/1472 (51.8)	389/865 (45.0)	0.002
*Residence, n/N (%)*				0.282
Changhua County	1433/2337 (61.3)	910/1472 (61.8)	523/865 (60.5)	
Changhua City	464/2337 (19.9)	299/1472 (20.3)	165/865 (19.1)	
Others	440/2337 (18.8)	263/1472 (17.9)	177/865 (20.5)	
*Outpatient clinic/N (%)*				< 0.001
Division of Gastroenterology	374/2337 (16.0)	293/1472 (19.9)	81/865 (9.4)	< 0.001
Department of Internal Medicine	668/2337 (28.6)	462/1472 (31.4)	206/865 (23.8)	< 0.001
Department of Surgery	536/2337 (22.9)	321/1472 (21.8)	215/865 (24.9)	0.101
Other department in the hospital	499/2337 (21.4)	236/1472 (16.0)	263/865 (30.4)	< 0.001
Referral or unknown	260/2337 (11.1)	160/1472 (10.9)	100/865 (11.6)	0.656

### Results of different retrieval strategies

Table 2 summarizes the results of different retrieval strategies. As found in the initial chart review and the treatment records in the PharmaCloud, most of the patients did not undergo anti-HCV therapy because of either death or severe comorbidity. The remaining patients were approached by the electronic reminding system during their clinic visit, followed by telephone contact.

**Table 2:** Patient retrieval strategies and results

Patient retrieval methods	Telephone	Electronic Remind	SMS	Others^a^	p value
*Back group*	98	1349	7	18	p < 0.0001
*Not back group*	515	90	3	277	
Treated in other hospital	142	30		49	
Unable to contact	261	1	2	17	
Refused evaluation	80	23	1	9	
Deceased	9	1		45	
Co-morbidity	8	12		153	
Others	15	3		4	
*Total case number*	613	1419	10	295	

^a^Other methods of patient retrieval include initial chart review for patient comorbidity status, survival and PharmaCloud review for therapy outside the hospital

### Demographic characteristics of patients receiving the HCV RNA testing

Table 3 lists the demographic characteristics of the 1472 patients with HCV RNA testing. Most of them aged 60–80 years old. Patients who were older (≥ 80), were male, and had recent outpatient visit in a non-gastroenterology division had a high rate of positive HCV RNA testing. Subsequent anti-HCV therapy was initiated for 88.1% of patients who were tested positive for HCV RNA. The reasons for not initiating anti-HCV therapy in the hospital were as follows: patient refusal, severe comorbidity, therapy in other hospitals, and loss of subsequent patient contact.

**Table 3:** Comparison of characteristics of patients receiving HCV RNA testing

	All patient receiving HCV RNA testing (n = 1472)	Positive HCV RNA (n = 621)	Negative HCV RNA (n = 851)	p value
Age, year, median (IQR)	64 (54–73)	64 (54–75)	64 (55–72)	0.711
Age level, n/N (%)				0.012
< 60	541/1472 (36.8)	231/621 (37.2)	310/851 (36.4)	0.804
60–80	738/1472 (50.1)	291/621 (46.9)	447/851 (52.5)	0.036
≥ 80	193/1472 (13.1)	99/621 (15.9)	94/851 (11.0)	0.008
Gender, n/N (%)	762/1472 (51.8)	331/621 (53.3)	431/851 (50.6)	0.314
Residence, n/N (%)				0.382
Others	263/1472 (17.9)	101/621 (16.3)	162/851 (19.0)	
Changhua County	910/1472 (61.8)	393/621 (63.3)	517/851 (60.8)	
Changhua City	299/1472 (20.3)	127/621 (20.5)	172/851 (20.2)	
Outpatient clinic, n/N (%)				< 0.001
Division of Gastroenterology	293/1472 (19.9)	104/621 (16.7)	189/851 (22.2)	0.012
Department of internal medicine	462/1472 (31.4)	185/621 (29.8)	277/851 (32.5)	0.285
Department of surgery	321/1472 (21.8)	121/621 (19.5)	200/851 (23.5)	0.075
Referral or unknown	160/1472 (10.9)	121/621 (19.5)	39/851 (4.6)	< 0.001
Other department in the hospital	236/1472 (16.0)	90/621 (14.5)	146/851 (17.2)	0.192
Anti-HCV therapy provided, n/N (%)	547/1472 (88.1)	547/621 (88.1)		
Reason for not initiating anti-HCV therapy/N (%)		74/621 (11.9)		
Loss for contact		6/74 (8.1)		
Treated in other hospital		7/74 (9.5)		
Refusal of therapy		25/74 (33.8)		
Deceased		3/74 (4.1)		
Severe illness		24/74 (32.4)		
Others		9/74 (12.2)		

## Discussion

This single-center retrospective study reported the HCV microelimination results focusing on retrieving patients with HCV infection who were lost to follow-up in a hospital-based setting. Unfortunately, a significant proportion (61.78%) of patients who were positive for HCV antibody was not evaluated for HCV therapy in the hospital. Through the project, a patient retrieval rate of 62.99% and the subsequent HCV treatment rate of 88% were achieved.

Screen All and Treat All strategy is ideal [15, 16] but not cost effective to eliminate HCV in most clinical settings. In the United States, only 20% of the 3.5 million HCV-infected patients were screened by serological tests, with only 27% of them had HCV RNA testing, and finally, only 9% of the total pool was successfully treated [17]. Another study from the Swedish National Patient Register found that two-thirds of the 29,217 HCV-infected patients need further HCV care. Effective identification of patients with chronic HCV infection is the first step toward HCV elimination and is beneficial for clinicians and public health officials with limited financial support and lack of screening manpower. Screening for high-risk subpopulations, such as injection drug users [7], prisoners [8], HIV-infected patients [2, 9], or dialysis patients [3], has been effective as the first step toward HCV elimination. Before the instruction of current direct oral anti-viral therapy, a low cure rate of 60%–80% and significant side effects from treatment related factor may account for the low HCV evaluation rate [18]. Patient factors such as unawareness of the disease, and lack of access to medical care, and healthcare system factors such lack of knowledge of the healthcare professionals and/or lack of interest for referral [19] account for the high percentage of HCV-infected patients without referral for further confirmatory testing or treatment in the hospital.

One way to help eliminate HCV infection is by utilizing an electronic reminding system, which reportedly can increase the referral rate from 17 to 65% of patients for their subsequent therapy [10, 14, 19]. Considering that our study had already identified the HCV population, our hospital conducted the project “Retrieval of Lost Patients of the Hospital,” which aimed to eliminate hepatitis C infection in the hospital as an initial step toward HCV elimination. Our study found that 61.78% of the hospital patients were uninvestigated for HCV care; nonetheless, two-thirds of them can be reached using an electronic reminding system, and more patients were retrieved via telephone call.

A similar patient retrieval approach was reported in the Netherlands and Spain (Table 4). Beekmans et al. [20] reported that 30.1% of patients with hepatitis C were lost to follow-up for hospital care and only three of the 150 candidate patients were re-evaluated for treatment in 2018. Furthermore, Patricia et al. [21] conducted the REACH study to retrieve patients with positive HCV diagnostic test results from four hospitals in Utrecht in 2019, and they obtained a patient retrieval rate of 17.4%. A recent interventional study from Spain [22] retrieved patients who were lost to follow-up through several phone calls and emails, and the retrieval rate was 46%.

**Table 4:** List of studies focusing on the retrieval of lost-to-follow-up patients for HCV micro-elimination in the literature

Year	Country	Candidate for retrieval/HCV Ab (+) patient	Retrieved patient/candidate for retrieval	Positive HCV RNA/tested patient	HCV therapy/positive HCV RNA	References
018	Holland	150/499 (30.1%)	4/20 (25%)	3/4 (75%)	3/3 (100%)	Beekmans et al. [20]
2019	Netherland	269/1913 (14.1%)	47/269 (17.4%)	42/47 (89.4%)	42/42 (100%)	Kracht et al. [21]
2020	Netherland	308/689 (44.7%)	90/308 (29%)	19/34 (55.9%)	12/19 (61.2%)	Heil et al. [27]
2021	Spain	530/1330 (39.8%)	244/530 (46%)	153/244 (62.7%)	141/153 (92.2%)	Guerra Veloz et al. [22]
2021	Taiwan	2337/3783 (61.8%)	1472/2337 (62.9%)	621/1472 (42.2%)	547/621 (88.1%)	Present study

In our study, the patient retrieval rate was high, owing to the use of both in-hospital “passive” approach (i.e., physicians are systematic made aware through diverse electronic reminding systems and reflex anti-HCV testing) and out-hospital “active” approach (i.e., contacting patients directly by phone and arranging schedules for one-step HCV testing to increase the test uptake). Hence, a combined approach is required to maximize the retrieval rate of patients who were lost to follow-up, especially when COVID-19 has significant impact for patient retrieval in the health care system during 2020 [23, 24]. In addition, multiple education activities for patients or health professionals from the government part and hospital part during the period explains our high patient retrieval rate. As reported in a previous study, older patients and patients with clinic visit in other departments are more difficult to retrieve [20, 22, 25], and further interventional measurements, such as education for both patients and health professionals, may further increase the patient retrieval rate and contribute to the goal of HCV elimination.

Meanwhile, the current study has several limitations. First, this retrospective study enrolled patients over a 1-year period in a single institution, with no standardized protocol to record the patients’ baseline characteristics for further analysis to enhance patient acceptance. Second, we encouraged patients during telephone call to undergo HCV testing and treatment. We failed to thoroughly investigate the reasons for patient refusal during telephone call; possible reasons include unstable housing or financial resources, concomitant psychiatric disease, or concern for COVID-19 infection in the hospital [26].

## Conclusions

This HCV-targeted callback approach identified a significant proportion of patients with positive HCV antibody that remained unevaluated for HCV RNA testing or therapy in the hospital. Therefore, this approach is feasible and effective in achieving HCV microelimination.

## Data Availability

The datasets used and/or analyzed during the current study are available from the corresponding author on reasonable request.
